# MELK promotes HCC carcinogenesis through modulating cuproptosis-related gene DLAT-mediated mitochondrial function

**DOI:** 10.1038/s41419-023-06264-3

**Published:** 2023-11-11

**Authors:** Zhipeng Li, Huaxin Zhou, Xiangyu Zhai, Lin Gao, Mengfan Yang, Baokun An, Tong Xia, Gang Du, Xiaoming Li, Wei Wang, Bin Jin

**Affiliations:** 1 Department of Hepatobiliary Surgery, The Second Hospital of Shandong University, Jinan, China; 2 The Second Clinical Medical School of Shandong University, Jinan, China; 3 Organ Transplant Department, Qilu Hospital of Shandong University, Jinan, China; 4 Department of General Surgery, Shandong Second Provincial General Hospital, Shandong Provincial ENT Hospital, Jinan, China; 5 Medical integration and practice center of Shandong University, Jinan, China

**Keywords:** Cancer metabolism, Targeted therapies

## Abstract

Cuproptosis caused by copper overload is mediated by a novel regulatory mechanism that differs from previously documented mechanisms regulating cell death. Cells dependent on mitochondrial respiration showed increased sensitivity to a copper ionophore elesclomol that induced cuproptosis. Maternal embryonic leucine zipper kinase(MELK) promotes tumorigenesis and tumor progression through the PI3K/mTOR pathway, which exerts its effects partly by targeting the pyruvate dehydrogenase complex(PDHc) and reprogramming the morphology and function of mitochondria. However, the role of MELK in cuproptosis remains unclear. Here, we validated that elevated MELK expression enhanced the activity of PI3K/mTOR signaling and subsequently promoted Dihydrolipoamide S-Acetyltransferase (DLAT) expression and stabilized mitochondrial function. This regulatory effect helped to improve mitochondrial respiration, eliminate excessive intracellular reactive oxygen species (ROS), reduce intracellular oxidative stress/damage and the possibility of mitochondria-induced cell fate alternations, and ultimately promote the progression of HCC. Meanwhile, elesclomol reduced translocase of outer mitochondrial membrane 20(TOM 20) expression and increased DLAT oligomers. Moreover, the above changes of MELK to HCC were abolished by elesclomol. In conclusion, MELK enhanced the levels of the cuproptosis-related signature(CRS) gene DLAT (especially the proportion of DLAT monomer) by activating the PI3K/mTOR pathway, thereby promoting elesclomol drug resistance, altering mitochondrial function, and ultimately promoting HCC progression.

## Introduction

Primary liver cancer is the sixth most commonly diagnosed cancer and the third leading cause of cancer mortality globally [1]. Hepatocellular carcinoma (HCC) comprises about 80% of primary liver cancer and has recently received considerable attention due to its rapidly increasing mortality rates [1, 2]. Although the treatment of HCC has progressed greatly over the last decade, the prognosis of this patient population remains dismal [3], partly because most HCC patients present with advanced-stage cancer at diagnosis when surgical or locoregional treatments are not feasible [1, 4]. Furthermore, it is a heterogeneous disease with limited response to radiotherapy and high chemoresistance [5]. Besides, although multikinase inhibitors or immune-checkpoint inhibitors (ICIs) have been successfully applied in treating HCC, the survival benefits of these agents for HCC patients remain disappointing [6]. Thus, further studies on HCC progression mechanisms are warranted for developing new therapeutic approaches.

Maternal embryonic leucine zipper kinase (MELK) is a member of the Snfl/AMPK family of serine/threonine kinase [7]. *MELK* is a novel oncogene that has been associated with multiple cellular functions, including carcinogenesis [8], proliferation [9, 10], apoptosis [11], stemness [12], and metabolism [13] and has drawn much attention in the field of cancer biology, stem cells, and metabolism recently [14, 15]. Moreover, the critical role of mitochondria in defining tumor stem stemness and tumor fate has been increasingly recognized [16, 17]. Recent studies have shown that MELK contributes to tumorigenesis and tumor progression through activation of the PI3K/mTOR cascade [18, 19]. Overexpression of MELK promoted tumor proliferation, migration, stemness, and Akt/mTOR signaling activity [20]. Alterations in cellular metabolism distinguish liver cancer cells from normal healthy cells, which is considered one of the ten hallmarks of cancer [21]. Energy production alterations help cancer cells to maintain high proliferation rates [22]. Therefore, targeting altered tumor metabolism might be a potential approach for tumor therapy [23].

The PI3K/mTOR signaling pathway has been associated with the pathogenesis of various tumors and mitochondrial metabolism [23, 24]. Current evidence suggests that PI3K promotes tumor cell progression by reprogramming the morphology and function of mitochondria [23]. The PI3K/mTOR signaling pathway has been shown to activate the pyruvate dehydrogenase complex (PDHc) by targeting mitochondrial proteins [25]. PDHc is a critical mitochondrial enzyme complex that promotes the conversion of pyruvate to generate acetyl-CoA and its entry into the tricarboxylic acid (TCA) cycle [26]. DLAT (Dihydrolipoamide S-Acetyltransferase), an essential component of mitochondrial PDHc, encodes the E2 subunit of PDHc [27]. The dysregulated expression of DLAT could impair PDHc activity and metabolic reprogramming and impair cellular stemness severely [27]. *DLAT* has been documented as an oncogene in various tumors [28, 29] and a novel cuproptosis-related signature gene [30]. *MELK* and the cuproptosis-related signature (CRS) gene *DLAT* have been validated as prognostic biomarkers for HCC patients [12, 29]. However, the role of MELK and DLAT in HCC progression, especially in HCC metabolism, remains obscure.

Our study found that the overexpression of MELK regulated the mitochondrial function and promoted HCC progression. We further found that MELK regulated the mitochondrial function of HCC cells by upregulating the expression of the CRS gene *DLAT*, enhancing the biological activity of HCC. Mechanistically, the PI3K/mTOR signaling pathway was responsible for the DLAT-mediated regulation of MELK on mitochondrial function in HCC cells. Notably, this study provides hitherto undocumented evidence on the role of MELK in HCC cell progression through CRS gene-mediated mitochondrial function changes, providing a promising therapeutic target for HCC therapy.

## Materials and methods

### Patient and ethical approval

The public databases were processed and visualized in Supplementary Table 1 and 2. A total of 106 Chinese patients with HCC underwent surgical resection at The Second Hospital of Shandong University from 2015.11 to 2021.10 (Supplementary Table 3). All patient materials used for research purposes were obtained after obtaining informed consent. The study protocol was approved by the Ethics Committee of The Second Hospital of Shandong University.

### Cells and agents

Human HCC cell lines HepG2, Hep3B, Huh7, and PLC/PRF/5 were obtained from the Cell Bank of the Chinese Academy of Sciences (Shanghai, China). All cell lines were cultured in DMEM (Thermo Fisher Scientific, Waltham, MA, USA) supplemented with 10% FBS (Thermo Fisher Scientific) and 1% penicillin/streptomycin(Hyclone, SV30010) at 37 °C under 95% air and 5% CO_2_.

All cell lines were confirmed by short tandem repeat (STR) analysis, and mycoplasma detection was conducted before performing any experiments. More detailed information on antibodies and reagents is provided in Supplementary Table 4.

### Tissue microarray and immunohistochemistry

The tissue microarrays (TMA) were constructed using paraffin-embedded tissues, as previously reported [31]. The histological features of all samples were identified by hematoxylin and eosin (HE) staining before immunohistochemistry (IHC).

For IHC, the tissue slides were de-paraffinized and then submerged in EDTA (pH = 9) buffer for antigen retrieval. Tissue sections were incubated with primary antibodies of MELK (1:200) or DLAT (1:100) at 4°C overnight. Then, the subsequent operations were performed as mentioned in our previous study [31].

The IHC score was quantified using QuantCenter software based on the staining intensity score and the percentage of stained cells as follows: IHC score = (percentage of weak intensity cells × 1) + (percentage of weak intensity cells × 2) + (percentage of weak intensity cells × 3). Based on the optimal cut-off value determined by receiver operating characteristic (ROC) curves, the cohort was divided into low and high expression levels of candidate biomarkers.

### RNA extraction and qPCR

Total RNAs were extracted from fresh clinical tissues or cells according to protocols using TRIzol reagent (Thermo Fisher), and cDNA synthesis was performed using a reverse transcriptase kit (TOYOBO, Japan) following the protocols. Quantitative PCR (qPCR) was performed as previously reported [31, 32]. The 2^−ΔΔCt^ method was used for comparison between groups. The primers used in qPCR are listed in Supplementary Table 5.

### Western blot and analysis

Tissue and total cell proteins, extracted with RIPA buffer (Solarbio Science, Beijing, China) supplemented with 1% PMSF (Beyotime, Shanghai, China)and 1% PI (Phosphatase Inhibitor, Solarbio), were analyzed by western blot. Protein bands were quantified using the ImageJ software.

### Transfection and stable cell lines

MELK knockdown or overexpression was induced by constructing the corresponding lentiviruses (GenePharma). For optimal transfection efficiency, the MELK overexpression transfection was repeated twice, as indicated by lv-MELK-1 and lv-MELK-2, respectively. Other transfections of Huh7, Hep3B, and MIHA cells were performed using Lipofectamine 2000 (Thermo Fisher). Puromycin (4 μg/ml) was used for the stable cell lines selected. The related sequences are listed in Supplementary Table 6.

### Cell proliferation and colony formation assays

CCK-8 (Dojindo, Kumamoto, Japan) assays were performed to assess cell proliferation according to the manufacturer’s recommendations. Briefly, 3000–5000 cells were plated into 96-well plates and incubated for 1–5 days.

For the colony formation assay, cells were seeded in 12-well plates (10,000 cells/well) and cultured in a complete medium. After 2 weeks, visible colonies were fixed with methanol and stained with crystal violet solution (0.5%) at room temperature for 15 minutes.

### Transwell assays

In all, 5–10 × 10^4^ cells were seeded into the upper chambers with or without matrigel coated(diluted at 1:6 with DMEM; Corning), and the lower chambers were added with 600 μL medium with 20% FBS. After 24–36 h incubation, the cells on the lower surface were fixed with methanol and stained with 0.5% crystal violet (Beyotime) at room temperature for 30 min. Cells in five random visual fields were photographed and quantified.

### Soft agar colony forming assay

The soft agar colony formation assay was performed for 3D sphere culture, as previously reported [31, 32]. All experiments were performed in triplicates.

### Immunofluorescence assay

For IF, cells were seeded on chamber slides in 24-well plates and cultured at 37 °C with 5% CO_2_. The cells were treated with 4% paraformaldehyde and 0.5% Triton X-100 to fix and permeabilize them, respectively, prior to overnight incubation with the primary antibody at a dilution of 2.5% serum. Then, cells were incubated with secondary antibodies goat anti-rabbit Alexa Fluor 594 (Invitrogen, Cat #A-21207) and anti-mouse Alexa Fluor 488 (Invitrogen, Cat#A-21202) prior to DAPI staining. Images were taken using laser scanning confocal microscopy (Carl Zeiss, LSM 800). Image-ProPlus was used to analyze Pearson’s correlation coefficients.

### Quantification of mitochondrial membrane potential

Cells with the indicated gene modifications or treated with reagents were seeded into 24-well plates. Afterward, cells were incubated with JC-1 (Invitrogen) to observe changes in the mitochondrial membrane potential according to the manufacturer’s instructions. The JC-1 green monomer intensity was measured by the Biotek ELX 800 microplate reader. Images of JC-1 green monomer and red dimer (J-aggregates) were captured using an Olympus fluorescence microscope (Olympus, Tokyo, Japan).

### ATP and reactive oxygen species measurements

1–3 × 10^5^ cells were seeded in 12-well plates and cultured for predefined periods. The ATP concentration in cells was determined using the ATP assay kit (Beyotime, Shanghai, China) according to the manufacturer’s instructions. The intracellular reactive oxygen species (ROS) concentration was measured using the DCFH-DA probe (Beyotime, Shanghai, China) [33]. Fluorescence imaging photographs were obtained using a fluorescence microscope (Olympus, Tokyo, Japan).

### Measurement of OCR

The Seahorse XFp Flux Analyzer (Seahorse Bioscience, Agilent) was used to detect the oxygen consumption rate (OCR). Briefly, 1–3 × 10^4^ cells were seeded into the 96-well XF Seahorse plate according to the attached protocols. The cells were cultured in XF base medium at 37 °C and were sequentially treated with Oligomycin (1 µM), FCCP (1 µM), and Rotenone/Antimycin A (0.5 µM) at predefined time points. The Seahorse XFp software was used to measure the OCR data.

### Xenograft models

Nude mice (BALB/c, female, 5 weeks of age, 16–22 g) were purchased from Vital River Laboratory Animal Technology Company Co., Ltd (Beijing, China). HCC cells were injected subcutaneously into the flanks of the nude mice (*n* = 6/group). Mice were examined every 3 days for tumor growth, and tumor diameter was measured with a caliper. Mice were randomly separated into groups when their tumor initially forms (~150 mm^3^). 3 to 4 weeks after tumor formation, mice were sacrificed. The tumor volume (V) was calculated using the following formula: *V* = (Length × Width ^2^)/2.

For the in vivo metastasis model, 4 × 10^5^ stable high MELK-expressing Huh7 cells were injected into the tail vein of each nude mouse treated with or without Elesclomol (10 mg/kg s.c.). The tumor burden of mice was quantified by measuring radiant efficiency using Living Image Software (IVIS Imaging Systems). The growth status of the mice was examined every 3 days, and the body weights were measured to assess the tumor burden. The number of tumor nodules on the organs was confirmed by HE staining based on conventional tissue and nuclear atypia criteria. All animal experiments were approved by the Medical Ethics Committee of The Second Hospital of Shandong University.

### Statistical analysis

SPSS17.0 (IBM, Chicago, IL, USA) and GraphPad Prism 5.0/8.0 (GraphPad Prism Software, San Diego, CA, USA) were used for statistical analysis. ImageJ software (National Institutes of Health, Bethesda, MD) was used for western blot analysis. The chi-square test was used to assess the correlation between MELK/DLAT and clinicopathological features. The correlation between the IHC score and *MELK/DLAT* was analyzed using Pearson’s correlation test. The plotting and statistical analysis of the survival curves were performed by the Kaplan-Meier method and the log-rank test, respectively. The cox-regression model was used for multivariate analysis, and independent prognostic factors were screened. Statistical comparisons of two paired groups or multi-groups were performed using t-test, one-way or two-way ANOVA. P value < 0.05 was statistically significant.

## Results

### Expression and clinical significance of MELK in HCC

**Fig. 1:**
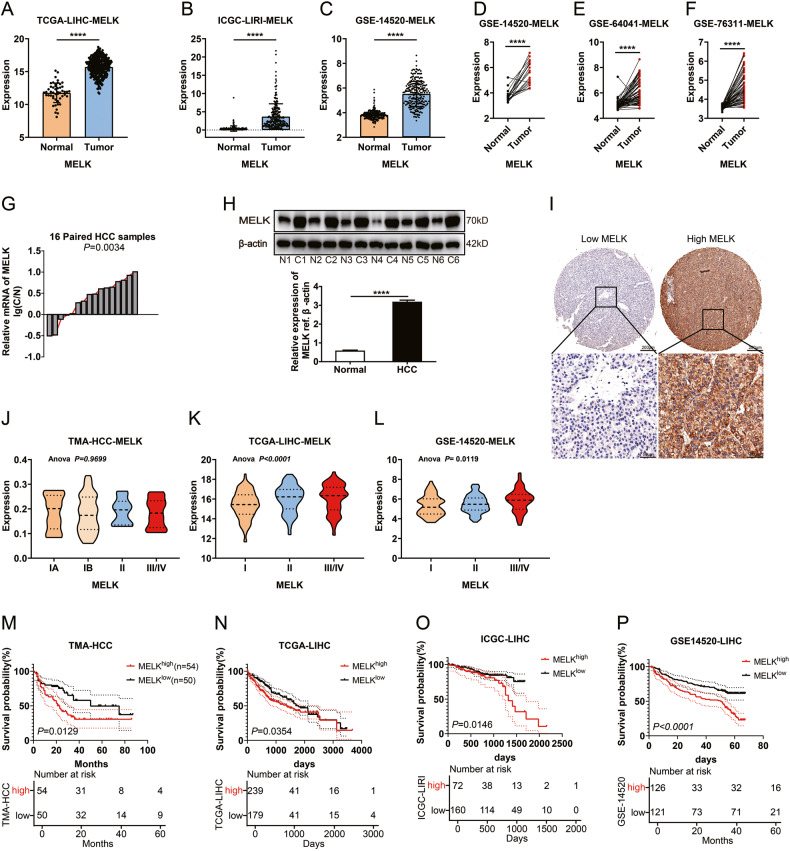
Expression and clinical significance of MELK in HCC. **A**–**F** Amplification of *MELK* is common in TCGA and GEO provisional HCC cohorts. The expression of *MELK* was detected with qPCR in 16 pairs of HCCs and adjacent tissues (**G**) and with WB in six randomly-selected pairs of HCC tissues (**H**). **I** Representative images of immunohistochemical staining for low/high expression of MELK in the tissue microarray (top: ×100 magnification, scale bar, 200 μm; bottom: ×400 magnification, scale bar, 50 μm). The expression of MELK in HCC patients with different clinical stages in TMA (**J**), TCGA (**K**), and GEO (**L**) provisional HCC cohort. The prognostic significance of MELK in TMA (**M**), TCGA-LIHC (**N**), ICGC-LIRI (**O**), and GSE14520 (**P**) cohorts. The *p* values were analyzed by Student’s *t* test and one-way or two-way ANOVA. The Kaplan–Meier method was used for prognosis analysis. Data were from at least three independent experiments and shown as the mean ± SEM ****P < 0.0001.

Previous studies reported that MELK was significantly upregulated in multiple human cancers and correlated with poor prognosis [12]. In the present study, we assessed MELK expression and explored its significance in HCC. As shown in Fig. 1A-F, *MELK* was upregulated in HCC compared with adjacent normal tissues. We further quantified the expression of *MELK* in HCC and their corresponding normal tissues with qPCR and WB and found that MELK was substantially upregulated in HCC (Fig. 1G, H). IHC of MELK was performed on 106 HCC tissues, dividing the cohort into high or low MELK expression subsets (Fig. 1I). Importantly, *MELK* expression increased in some cohorts with advanced clinical staging (Fig. 1J–L). Moreover, MELK upregulation was significantly correlated with positive tumor size and advanced TNM/histological stage (Supplementary Table 1–3), suggesting that MELK may promote HCC progression. Survival analysis revealed that high expression of MELK was associated with poor outcomes (Fig. 1M and Supplementary Table 3). Similarly, the high *MELK* expression group in the TCGA-LIHC, ICGC-LIRI, and GSE14520 datasets showed a significantly worse prognosis (Fig. 1N–P and Supplementary Fig. 1A), suggesting that MELK is a potential prognostic biomarker for HCC.

### MELK promoted HCC progression in vitro

**Fig. 2:**
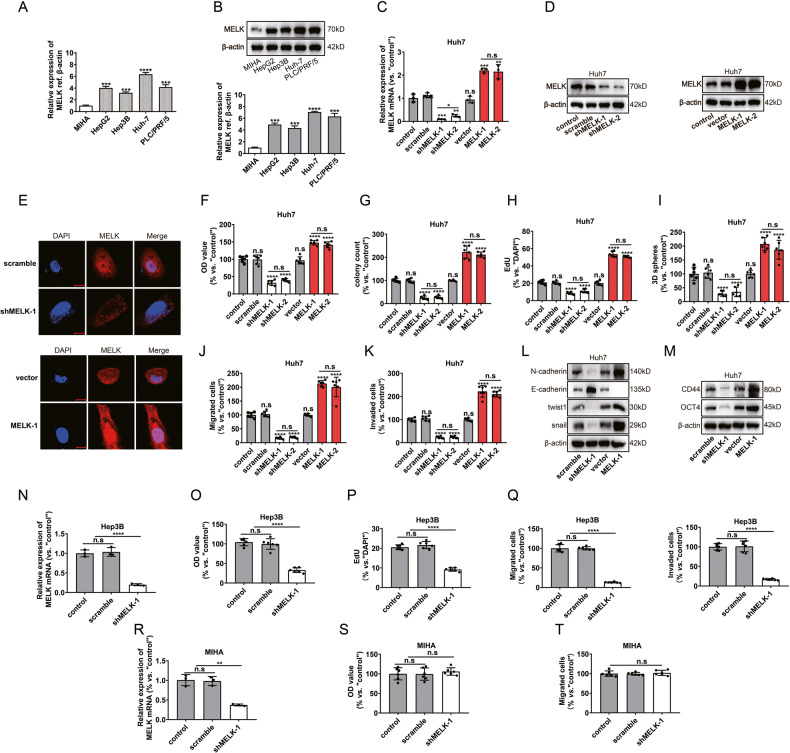
MELK promoted HCC cell progression in vitro. **A**, **B** The mRNA and protein expressions of MELK in normal human hepatocytes (MIHA) and different HCC cell lines: HepG 2, Hep 3B, Huh7, and PLC/PRF/5. After MELK expression was silenced or overexpressed in Huh7 cells: MELK expression was detected by qPCR (**C**), WB (**D**) and immunofluorescence (**E**); Cell proliferation was evaluated with CCK-8 assay (**F**), colony formation assay (**G**) and EdU assay (**H**); Stemness was evaluated with the 3D sphere formation assay (**I**); Cell migration and invasion were investigated with transwell assays (**J**, **K**); Biomarkers of EMT and stemness were detected by WB (**L**, **M**). After *MELK* knockdown in the HCC cell line Hep3B and the normal human hepatocytes MIHA: *MELK* expression was detected by qPCR (**N**, **R**); Cell proliferation was evaluated with CCK-8 assay (**O**, **S**) and EdU assay (**P**); Cell migration and invasion were investigated with transwell assays (**Q**, **T**). Data were shown as mean ± SEM. n.s. represents not significant. *P < 0.05, **P < 0.01, ***P < 0.001 and ****P < 0.0001 as calculated by the one-way or two-way ANOVA. Scale bar: 10 µm.

Next, the Broad Institute DepMap website (https://depmap.org) was used to analyze the expression and the effects of *MELK* in various HCC cell lines (Supplementary Fig. 1B). Next, we confirmed that *MELK* expression at the mRNA (Fig. 2A) and protein (Fig. 2B) levels were upregulated in the HCC cell lines. Conversely, low MELK expression was detected in normal human hepatocytes (MIHA) (Fig. 2A, B). The above results indicate that MELK is overexpressed in HCC. In subsequent experiments, MELK was silenced or overexpressed in Huh 7 cells first (Fig. 2C–E). CCK-8, colony formation, and EdU assays showed that MELK knockdown significantly impaired proliferation, while overexpression of MELK promoted HCC proliferation (Fig. 2F–H and Supplementary Fig. 1D, E). To verify the roles of MELK in HCC progression, the effects of MELK on cell stemness, migration, and invasion (Supplementary Fig. 2A–G) were investigated, given that MELK has been associated with these activities [14, 15]. Sphere formation and transwell assays showed that MELK promoted the stemness, migration, and invasion of Huh7 cells (Fig. 2I–M and Supplementary Fig. 1F, G).

Furthermore, we investigated the potential role of MELK in other HCC cells by infecting Hep3B with *MELK*-shRNA lentivirus. We observed significant inhibition of cell proliferation, migration, and invasion in Hep3B cells with decreased *MELK* mRNA expression.(Fig. 2N–Q and Supplementary Fig. 2H, I). To investigate the potential function of MELK in non-cancerous hepatocytes, normal human hepatocytes (MIHA) were cultured and infected with the *MELK*-shRNA. In the stably transfected cells, robust *MELK* mRNA downregulation was detected (Fig. 2R). Importantly, shRNA-induced *MELK* silencing did not significantly inhibit the activity of normal hepatocytes (Fig. 2S, T and Supplementary Fig. 2J). Overall, the above results indicate the cancer cell-specific effects of MELK.

### MELK promotes mitochondrial function in vitro

**Fig. 3:**
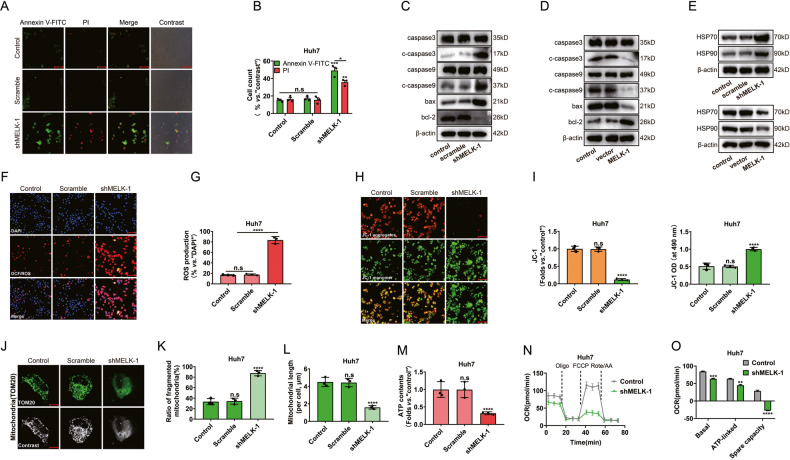
MELK promotes mitochondrial function in vitro. After *MELK* knockdown in Huh7 cells: Cell status was detected by immunofluorescence (**A**, **B**); Biomarkers of mitochondria-associated programmed death were detected by WB (**C**, **D**). MELK silencing significantly increased the expression of HSP 70 and HSP 90 (**E**) and the intensity of DCF/ROS (**F**, **G**). Changes of JC-1 green monomer and JC-1 red aggregates were detected by fluorescence microscope (**H**, **I** left). The JC-1 green monomer intensity was measured by Biotek ELX 800 microplate reader (**I** right). After MELK knockdown, mitochondrial dynamics were detected by immunofluorescence (**J**–**L**); ATP contents and OCR of cells were measured by the Seahorse XFp Flux Analyzer (**M**–**O**). Data were shown as mean ± SEM. n.s. represents not significant. *P < 0.05, **P < 0.01, ***P < 0.001 and ****P < 0.0001 as calculated by the one-way or two-way ANOVA. Scale bar:100 µm (**A**, **F**, **H**).

An increasing body of evidence suggests that the stabilization of mitochondrial function plays a critical role in maintaining tumor stemness and tumor progression [16, 17]. Next, we evaluated the effect of MELK on mitochondria-related functions. As shown in Fig. 3A, B, an increase in early apoptosis and necrosis was observed after MELK silencing. Meanwhile, the expression of mitochondria-related apoptosis markers was increased by shRNA and suppressed by overexpressing *MELK-1* (Fig. 3C, D). Importantly, the silencing of MELK increased the expression of the oxidative stress/damage proteins HSP 70 and HSP 90 (Fig. 3E). Besides, the DCF/ROS intensity increased significantly in *shMELK* cells, indicating ROS production and oxidative damage to the cells (Fig. 3F, G). In addition, MELK silencing impaired mitochondrial function, resulting in decreased mitochondrial membrane potential and mitochondrial depolarization, with the latter manifesting as the accumulation of JC-1 green monomers (Fig. 3H, I). Moreover, we found that mitochondrial dynamics were affected by MELK significantly, with changes observed in the number of mitochondrial fragments and mitochondrial length (Fig. 3J–L). Furthermore, MELK expression was related to the cellular ATP contents (Fig. 3M). It is well-established that mitochondrial respiration promotes substantial energy production in tumor cells and plays important roles in critical processes such as tumor stemness [34]. When the oxygen consumption rate (OCR) in cells was measured by the Seahorse Flux Analyzer (Fig. 3N), we found that MELK silencing led to a significant decline in the crux parameters of mitochondrial respiration, especially the spare respiratory capacity (Fig. 3O). This finding suggests that MELK is crucial for sustaining the biological function of tumor cells by regulating mitochondrial respiration. Overall, the dysregulation of mitochondrial function associated with MELK in tumor cells induced mitochondrial damage and cell death.

### MELK augments the expression of the CRS gene DLAT through the PI3K/mTOR signaling pathway

**Fig. 4:**
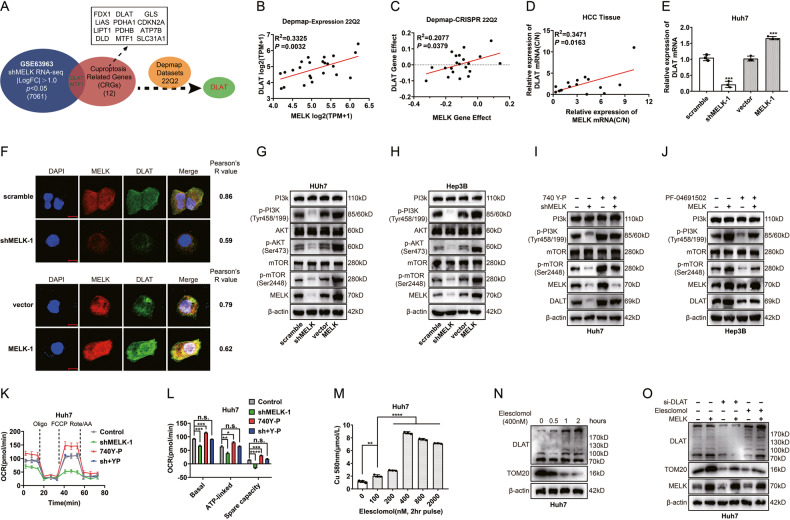
MELK augments the expression of the CRS gene *DLAT* through the PI3K/mTOR signaling pathway. **A** *DLAT* was filtered out as the only candidate gene among the datasets of sh*MELK*, cuproptosis-related signatures(CRS) genes and the datasets of DepMap. **B**–**D** Correlation of expression, gene effect and mRNA ratio between *MELK* and *DLAT*. The mRNA ratio was calculated by mRNA (tumor)/mRNA (adjacent tissue). **E**, **F** qPCR and immunofluorescence showed that *MELK* expression regulated the expression of *DLAT* in Huh7 cells. **G**, **H** WB showed that the PI3K signal pathway was regulated by MELK. **I**, **J** MELK-silenced or MELK-overexpressing Huh7 or Hep3B cells were incubated in PI3K agonist 740 Y-P (30 μM, 24 h) or PI3K inhibitor PF-04691502 (0.3 μM, 48 h), and DLAT expression was detected with WB. **K**, **L** Mitochondrial respiration was measured by OCR assay in Huh7 cells treated with MELK knockdown or/and 740 Y-P. **M** Intracellular copper contents with pulse treatment of elesclomol for 2 hr. **N** The expression of TOM20 and DLAT (monomer and oligomer) was detected by WB after treatment with elesclomol. **O** In MELK-overexpressed Huh7 cells, DLAT was knocked down or regulated by elesclomol, and the expression of TOM20 and DLAT were detected. n.s., *, **, *** and **** represented not signifificant, *P* < 0.05, <0.01, <0.001 and <0.0001, respectively. Analyzed data were from three independent experiments and shown as means ± SEM. Data were from three independent experiments and analyzed with the *t* test (**E**, **L**, and **O**). Spearman correlation analysis was performed in (**B**–**D**, **F**).

Recent studies have shown that cells dependent on mitochondrial respiration are more sensitive to a copper ionophore [30]. Moreover, copper can significantly reduce the spare respiratory capacity of tumor cells by directly targeting components of the TCA cycle, thereby promoting cuproptosis [30, 35]. This effect was similar to our findings following MELK silencing. Next, we sought to explore whether there was an association between MELK and cell cuproptosis. In our study, DLAT (LogFC: −1.19) and MTF1 (LogFC: −1.03) were screened from the datasets of *shMELK* RNA-seq and CRS [30] (Fig. 4A). After conducting an additional screening of two candidate genes with the assistance of the Broad Institute DepMap web portal, the cuproptosis-related gene *DLAT* was identified (Fig. 4B, C and Supplementary Fig. 3). Our qPCR results of HCC tissues also showed a certain positive correlation between *MELK* and *DLAT* (Fig. 4D). Interestingly, both qPCR and immunofluorescence assays showed that MELK regulated the expression of DLAT in Huh7 cells (Fig. 4E, F).

Studies have shown that MELK could promote the progression of tumor cells by activating the PI3K/mTOR signaling pathway [18, 19], catalyzing the PDHc [25], and reprogramming the morphology and function of mitochondria [23]. In the present study, WB analysis showed that MELK altered the phosphorylation levels of PI3K, Akt, and mTOR (Fig. 4G, H). To further evaluate the role of PI3K/mTOR signaling in MELK-mediated DLAT expression, we silenced MELK expression or/and stimulated PI3K/mTOR activation in Huh7 cells. We found that MELK knockdown or PI3K/mTOR activity activation altered the expression of DLAT (Fig. 4I). In contrast, the Hep3B cells that overexpressed MELK were subjected to treatment with the PI3K/mTOR signaling antagonist PF-04691502 (Fig. 4J). The findings demonstrated that MELK stimulated the expression of DLAT in hepatocellular carcinoma (HCC) cells through the activation of the PI3K/mTOR signaling pathway. (Fig. 4I, J). The above results suggest that MELK induced the expression of DLAT by activating the PI3K/mTOR signaling pathway. Due to its vital role in the pyruvate dehydrogenase complex, abnormal expression of DLAT can result in decreased PDHc activity and metabolic reprogramming, ultimately causing a significant decline in cellular stemness [27]. In vitro experiments were performed to evaluate the effect of MELK on mitochondrial respiration after inducing changes in DLAT through the PI3K/mTOR signal pathway. OCR assays demonstrated that MELK knockdown attenuated PI3K/mTOR-induced mitochondrial respiration, especially the spare respiratory capacity, in Huh7 cells (Fig. 4K, L). It has been established that the copper ionophore (elesclomol) induces cell cuproptosis by regulating mitochondrial respiration [30]. The results of the quantification of copper contents indicate that pulse treatment with various concentrations of elesclomol for 2 hours resulted in varying increments in intracellular copper levels (Fig. 4M). Next, the effect of elesclomol treatment on the expression of TOM20 (translocase of the outer mitochondrial membrane 20) and DLAT was examined. The results showed that elesclomol treatment reduced TOM 20 expression and increased DLAT oligomer (which inhibits the function of DLAT oncogene and leads to cuproptosis) (Fig. 4N). To further validate the role of DLAT in MELK-mediated mitochondrial function, we silenced DLAT expression or inhibited DLAT activity in MELK-overexpressing Huh7 cells (Fig. 4O). The findings indicated that the expression downregulation or the activity inhibition of DLAT impeded the expression of TOM20, which was induced by the overexpression of MELK.

### DLAT was highly upregulated in HCC and associated with a poor prognosis

**Fig. 5:**
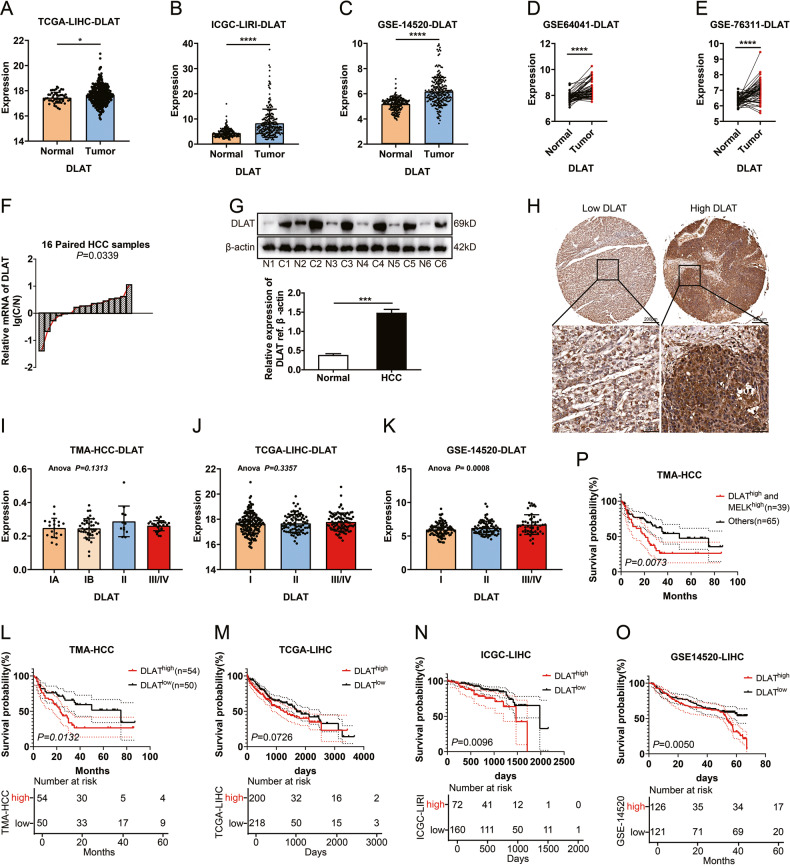
DLAT was highly expressed and associated with a poor prognosis in HCC. **A**–**E** Amplification of *DLAT* is common in TCGA and GEO provisional HCC cohorts. The expression of *DLAT* was detected with qPCR in 16 pairs of HCCs (**F**) and adjacent tissues and with WB in six randomly-selected pairs of HCC tissues (**G**). **H** Representative images of immunohistochemical staining for low/high expression of DLAT in the tissue microarray (top: ×100 magnification, scale bar, 200 μm; bottom: ×400 magnification, scale bar, 50 μm). The expression of *DLAT* in HCC patients with different clinical stages in TMA (**I**), TCGA (**J**), and GEO (**K**) provisional HCC cohorts. The prognostic significance of MELK in TMA (**L**, **P**), TCGA-LIHC (**M**), ICGC-LIRI (**N**), and GSE14520 (**O**) cohorts. The *p* values were analyzed by Student’s *t* test and one-way or two-way ANOVA. The Kaplan–Meier method was used for prognosis analysis. Data were from at least three independent experiments and shown as the mean ± SEM. n.s. represents not significant. ****P < 0.0001.

DLAT has been established to be an essential component of mitochondrial PDHc [27] and is a key CRS gene [30] that serves as an oncogene in multiple cancers [28, 29]. As shown in Fig. 5A–E, *DLAT* expression was significantly higher in HCC tissues than in normal tissues in some cohorts. qPCR and WB results showed that *DLAT* was highly upregulated in HCC (Fig. 5F, G). IHC of DLAT was performed on 106 HCC tissues, dividing the cohort into high or low DLAT expression subsets (Fig. 5H). Moreover, *DLAT* expression increased with more advanced clinical stages in GSE-14520 cohort, similar to *MELK* (Fig. 5I–K). Furthermore, high expression of DLAT was significantly associated with tumor size and advanced TNM/BCLC stage (Supplementary Table 1–3), suggesting that DLAT may promote HCC progression. Similarly, survival analysis showed high DLAT was associated with poor outcomes (Fig. 5L–O and Supplementary Table 1–3). Importantly, high expression of both MELK and DLAT was a more sensitive prognostic factor than MELK or DLAT alone (Fig. 5P).

### The cuproptosis-associated pathway was essential in MELK-mediated mitochondrial alterations

**Fig. 6:**
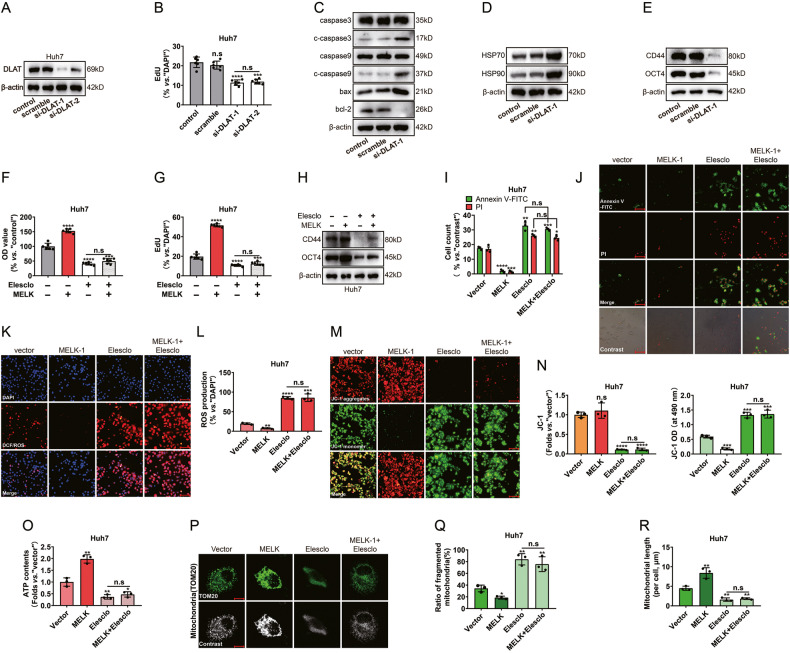
The cuproptosis-associated pathway was essential in MELK-mediated mitochondrial alterations. After silencing DLAT with siRNAs in the Huh7 cells: MELK expression was detected by WB (**A**); Cell proliferation was evaluated with EdU assay (**B**); Biomarkers of the mitochondria-associated programmed death, the HSP 70 and HSP 90 and the stemness were detected by WB (**C**–**E**). After treatment with or without elesclomol in MELK-overexpressed Huh7 cells: Cell proliferation was evaluated with CCK-8 assay (**F**) and EdU assay (**G**); Biomarkers of stemness were detected by WB (**H**); The mitochondria-associated programmed death (**I**, **J**), the intensity of DCF/ROS (**K**, **L**), the changes of JC-1 monomer/aggregates (**M**) and mitochondrial dynamics were detected by immunofluorescence (**P**, scale bar:10 µm); The JC-1 green monomer intensity was measured by a microplate reader (**N**); ATP contents of cells were measured by the Seahorse XFp Flux Analyzer (**O**). Data were shown as mean ± SEM. n.s. represents not significant. *P < 0.05, **P < 0.01, ***P < 0.001 and ****P < 0.0001 as calculated by the one-way or two-way ANOVA. Scale bar: 100 µm (**J**, **K**, **M**).

To further support the role of DLAT in HCC cells, *DLAT* knockdown was induced by si-RNAs (Fig. 6A). DLAT knockdown significantly inhibited Huh7 cell proliferation with a decreased EdU-positive nuclei ratio (Fig. 6B and Supplementary Fig. 4A). The *si-DLAT* significantly promoted the expression of mitochondria-related programmed death and the oxidative stress/damage proteins HSP 70 and HSP 90 in Huh 7 cells while inhibiting the expression of cell stemness markers (Fig. 6C-E). Elesclomol-induced cuproptosis was achieved by regulating the oligomerization of DLAT, followed by regulating mitochondrial respiration [30]. However, whether the cuproptosis pathway participated in MELK-mediated functional alterations of Mitochondria remains unclear. To detect the role of cuproptosis in MELK-induced mitochondria-associated HCC progression, MELK-overexpressing cells were treated with elesclomol. CCK8 and EdU assays showed that elesclomol attenuated MELK-induced proliferation in Huh7 cells (Fig. 6F, G and Supplementary Fig. 4B). Meanwhile, the expression of cell stemness markers was significantly altered (Fig. 6H). The apoptosis of MELK-overexpressing Huh7 cells was impaired after elesclomol treatment (Fig. 6I, J). MELK overexpression decreased ROS production, whereas elesclomol abolished this effect (Fig. 6K, L). Quantification of mitochondrial membrane potential and ATP contents showed that elesclomol significantly impaired MELK-induced cell JC-1 red aggregates and ATP contents (Fig. 6M–O). Moreover, mitochondrial dynamics were accompanied by significant changes with the above treatments (Fig. 6P–R).

### The cuproptosis-associated pathway was essential in MELK-induced HCC progression in vivo

**Fig. 7:**
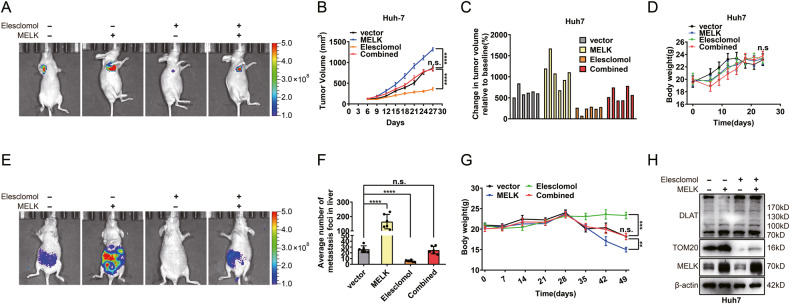
The cuproptosis-associated pathway was essential in MELK-induced HCC progression in vivo. **A** Xenograft models were established by subcutaneous injection of MELK-overexpressing Huh7 cells, elesclomol (10 mg/kg s.c.) was used to regulate DLAT status in vivo. Changes in subcutaneous xenograft tumors and body weight were observed (**B**–**D**). **E** Metastatic models were established by tail vein injection of MELK-overexpressing Huh7 cells, elesclomol (10 mg/kg s.c.) was used to regulate DLAT status in vivo. The tumor metastases were monitored by a live imaging system. The metastatic nodules in the liver were measured (**F**). And the changes in body weight of indicated groups of nude mice models were observed (**G**). **H** The expression of TOM20 and DLAT of tumors assessed via WB. Data were shown as mean ± SEM. n.s. represents not significant. *P < 0.05, **P < 0.01, ***P < 0.001 and ****P < 0.0001 as calculated by the one-way or two-way ANOVA.

**Fig. 8:**
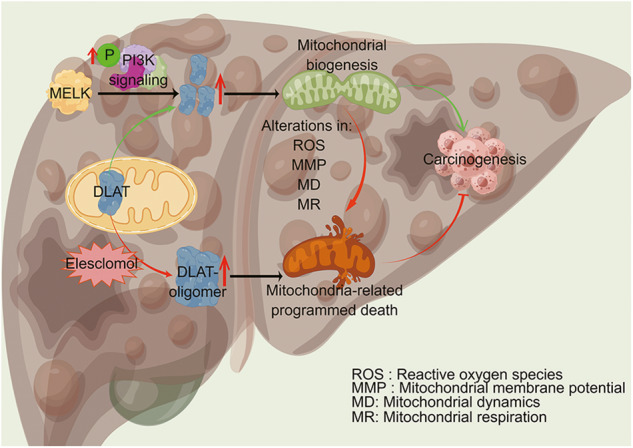
Schematic depiction of the mechanism by which MELK promotes HCC carcinogenesis through modulating DLAT-mediated mitochondrial function. MELK augments the expression of the CRS gene *DLAT* through the PI3K/mTOR signaling pathway and promotes mitochondrial function, which subsequently promotes the progression of HCC. Abnormal expression of MELK impaired mitochondrial function through alterations in ROS, MMP, MD and MR. The copper ionophore (elesclomol) increased DLAT oligomer (which inhibits the function of DLAT oncogene and leads to cuproptosis), inducing mitochondria-related programmed death, and subsequently inhibiting the progression of HCC. In all, evidence described above showed that the cuproptosis-associated pathway was essential in MELK-induced HCC progression. *Figure was drawn by Figdraw(WIWWAacf4a).

Experiments were conducted on nude mice to examine the impact of elesclomol on MELK-mediated HCC progression. The mice were treated with either MELK-overexpressing or/and elesclomol to further assess the role of elesclomol during this process. Xenografts were established with MELK-overexpressing stable cells with or without elesclomol treatment, showing that elesclomol significantly reduced tumor volume and weight, which were increased by MELK overexpression (Fig. 7A–C and Supplementary Fig. 4C). Meanwhile, there was no significant change in the body weight of nude mice, indicating the overall safety of elesclomol administration (Fig. 7D). In the metastatic model, the results showed that elesclomol substantially counteracted the promoting effect of MELK on HCC metastasis (Fig. 7E, F). The averaged number of foci in liver was significantly less in the elesclomol group than in the other groups, resulting in a more stable body weight (Fig. 7E–G). To further evaluate the mechanism of this treatment, a WB assay was performed for subcutaneous xenografts in nude mice. MELK overexpression increased the expression of TOM20, whereas the addition of elesclomol abolished this effect (Fig. 7I). Overall, the above results confirmed that the cuproptosis-associated pathway was essential in MELK-induced HCC progression in vivo (Fig. 8).

## Discussion

Hepatocellular carcinoma is one of the most common tumors and the third leading cause of cancer death globally. However, only a minority of early-stage HCC patients are indicated for surgical or locoregional treatments. Current evidence suggests that patients with advanced HCC, accounting for about 80% of all cases, generally have a less than 12% 5-year survival rate due to limited treatment options [36]. Although the treatment of HCC has progressed greatly in the last decade, the prognosis of patients with HCC remains far from satisfactory [3]. Considering the high heterogeneity of response in HCC patients, identifying biomarkers conducive to treatment remains a top priority.

Alterations in cellular metabolism distinguish liver cancer cells from normal healthy cells [21]. In this regard, metabolic reprogramming is one of the ten hallmarks of cancer that can affect cancer cell growth and survival [22]. Recent studies have shown that the stabilization of mitochondrial function plays a critical role in maintaining tumor stemness and tumor progression [16, 17]. Therefore, tumor metabolism has emerged as a potential target for therapy [23]. In the present study, we demonstrated that MELK enhanced the healthy status of mitochondria and promoted the progression of HCC. We further found that MELK enhanced the expression of the CRS gene *DLAT* by enhancing the activation of the PI3K/mTOR signaling pathway, thereby promoting the progression of HCC. Overall, DLAT is essential for maintaining the balance of mitochondrial dynamics and functional homeostasis. Our results reveal a novel mechanism by which MELK promotes HCC progression through CRS gene-mediated mitochondrial function changes.

MELK is a member of the Snfl/AMPK family of serine/threonine kinase [7]. It has been reported that MELK is significantly upregulated in multiple types of human cancer and associated with poor prognosis [12]. Moreover, MELK has been associated with multiple cellular functions, including carcinogenesis [8], proliferation [8, 9], apoptosis [11], stemness [12], and metabolism [13]. Recent studies have shown that MELK contributes to tumorigenesis and tumor progression through activation of the PI3K/mTOR cascade [18, 19]. The PI3K/mTOR signaling pathway has been documented to promote tumor cell progression by targeting the pyruvate dehydrogenase complex [25] and reprogramming the morphology and function of mitochondria [23].

Copper is an essential cofactor of enzymes in animals that may eventually lead to cell death even at moderate intracellular concentrations [30]. The liver is an important organ for copper metabolism in the body. It has been established that liver cirrhosis which increases susceptibility to HCC, shows a certain degree of copper accumulation compared with the healthy liver. Since copper has the potential to induce oxidative stress, changes in copper homeostasis can lead to multiple life-threatening diseases [30, 37]. Moreover, copper can significantly reduce the spare respiratory capacity of tumor cells by directly targeting components of the TCA cycle, thereby promoting cuproptosis [30, 35]. Cuproptosis caused by copper overload is mediated by a novel regulatory mechanism that differs from previously documented mechanisms regulating cell death [30]. Recent studies have shown that cells dependent on mitochondrial respiration showed increased sensitivity to a copper ionophore elesclomol that induced cuproptosis [30]. Besides, the significant inhibitory effect of elesclomol has been documented in a variety of cells, including cancer stem cells [38], drug-resistant cells [39], and cells with enhanced mitochondrial metabolism [40]. Interestingly, the above effects of cuproptosis on mitochondrial respiration are similar to MELK silencing in HCC. Therefore, we hypothesized that there could be an interplay between MELK and the cuproptosis pathway in HCC cells to promote HCC progression.

Moreover, an in-depth analysis of the datasets of *shMELK* combined with cuproptosis-related signatures and using data from the Broad Institute DepMap web portal and related experimental verification were performed. Ultimately, 1 key cuproptosis-related gene *DLAT* was identified. DLAT is an essential component of mitochondrial PDHc since it encodes the E2 subunit [27]. DLAT was confirmed to be an oncogene in various tumors [28, 29] and a novel cuproptosis-related signature gene [30]. Dysregulated expression of DLAT could impair PDHc activity and metabolic reprogramming and impair cellular stemness severely [27]. Elesclomol-induced cuproptosis is a crucial discovery in cancer research and treatment. Copper ionophore elesclomol [41, 42] has been considered an anti-cancer drug targeting mitochondrial metabolism. Elesclomol can promote the increase of DLAT oligomers, which account for their toxic effects and finally induce cuproptosis [30]. To explore the potential association between MELK and cell cuproptosis, subsequent experiments were performed. We validated that MELK was highly expressed in HCC tissues. Elevated MELK expression enhanced the activity of PI3K/mTOR signaling and subsequently promoted DLAT expression and stabilized mitochondrial function. This regulatory effect helped to improve mitochondrial respiration, eliminate excessive intracellular ROS, reduce intracellular oxidative stress/damage and the possibility of mitochondria-induced cell fate alternations, and ultimately promote the progression of HCC. Meanwhile, the results showed that elesclomol treatment reduced TOM 20 expression and increased DLAT oligomers. Moreover, the above changes of MELK to HCC were abolished by elesclomol in vitro and in vivo.

## Conclusions

In conclusion, high expression of MELK or/and DLAT is a poor prognostic indicator in HCC. The present study provides hitherto undocumented evidence that altered mitochondrial function induced by MELK may be involved in the carcinogenesis of HCC. In addition, we reveal that MELK enhanced the levels of the CRS gene *DLAT* by activation of the PI3K/mTOR signaling pathway, thereby promoting the elesclomol drug-resistant and altering mitochondrial function and ultimately accelerating the progression of HCC. These findings refine our understanding of the mechanism of MELK-induced carcinogenesis and suggest that targeting the MELK/DLAT-mediated mitochondrial pathway may be a potential strategy for treating HCC.

### Supplementary information

Supplemental Materials

Reproducibility checklist

## Data Availability

All the data generated or analyzed in this study are included in this published article and its additional files.
