# Correction: MELK promotes HCC carcinogenesis through modulating cuproptosis-related gene DLAT-mediated mitochondrial function

**DOI:** 10.1038/s41419-023-06367-x

**Published:** 2023-12-18

**Authors:** Zhipeng Li, Huaxin Zhou, Xiangyu Zhai, Lin Gao, Mengfan Yang, Baokun An, Tong Xia, Gang Du, Xiaoming Li, Wei Wang, Bin Jin

**Affiliations:** 1 Department of Hepatobiliary Surgery, The Second Hospital of Shandong University, Jinan, China; 2 The Second Clinical Medical School of Shandong University, Jinan, China; 3 Organ Transplant Department, Qilu Hospital of Shandong University, Jinan, China; 4 Department of General Surgery, Shandong Second Provincial General Hospital, Shandong Provincial ENT Hospital, Jinan, China; 5 Medical integration and practice center of Shandong University, Jinan, China

**Keywords:** Cancer metabolism, Targeted therapies

Correction to: *Cell Death and Disease* 10.1038/s41419-023-06264-3, published online 11 November 2023

After the completion of this study, we proceeded to implement an archiving initiative for the pertinent experimental raw data. However, while organizing the original data presented in Fig. 7, we found that a failed image had unintentionally been included in Fig. 7A due to the naming similarity among the original images.

New Fig. 7
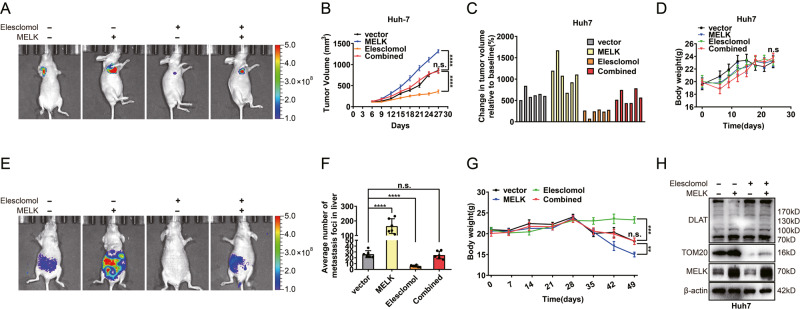


Original fig. 7
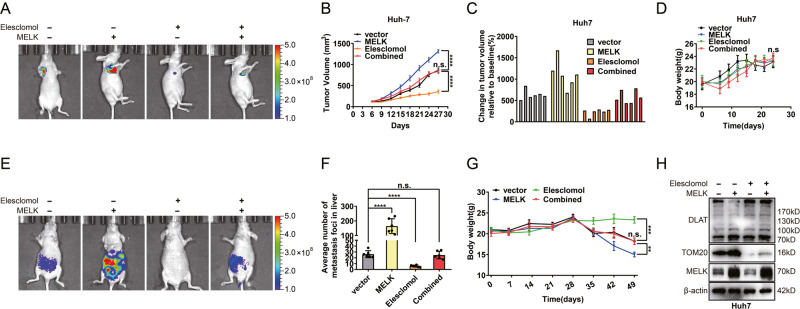


The original article has been corrected.

