# Supplementary material for: MELK promotes HCC carcinogenesis through modulating cuproptosis-related gene DLAT-mediated mitochondrial function
Source: Cell Death Dis. 2023 Nov 11;14(11):733. doi: 10.1038/s41419-023-06264-3 (PMC10638394; doi:10.1038/s41419-023-06264-3)
Supplement: Supplementary file 1 — Supplemental Materials [file 41419_2023_6264_MOESM1_ESM.pdf]

Supplemental Figure S1. MELK promoted HCC progression

supplemental-1

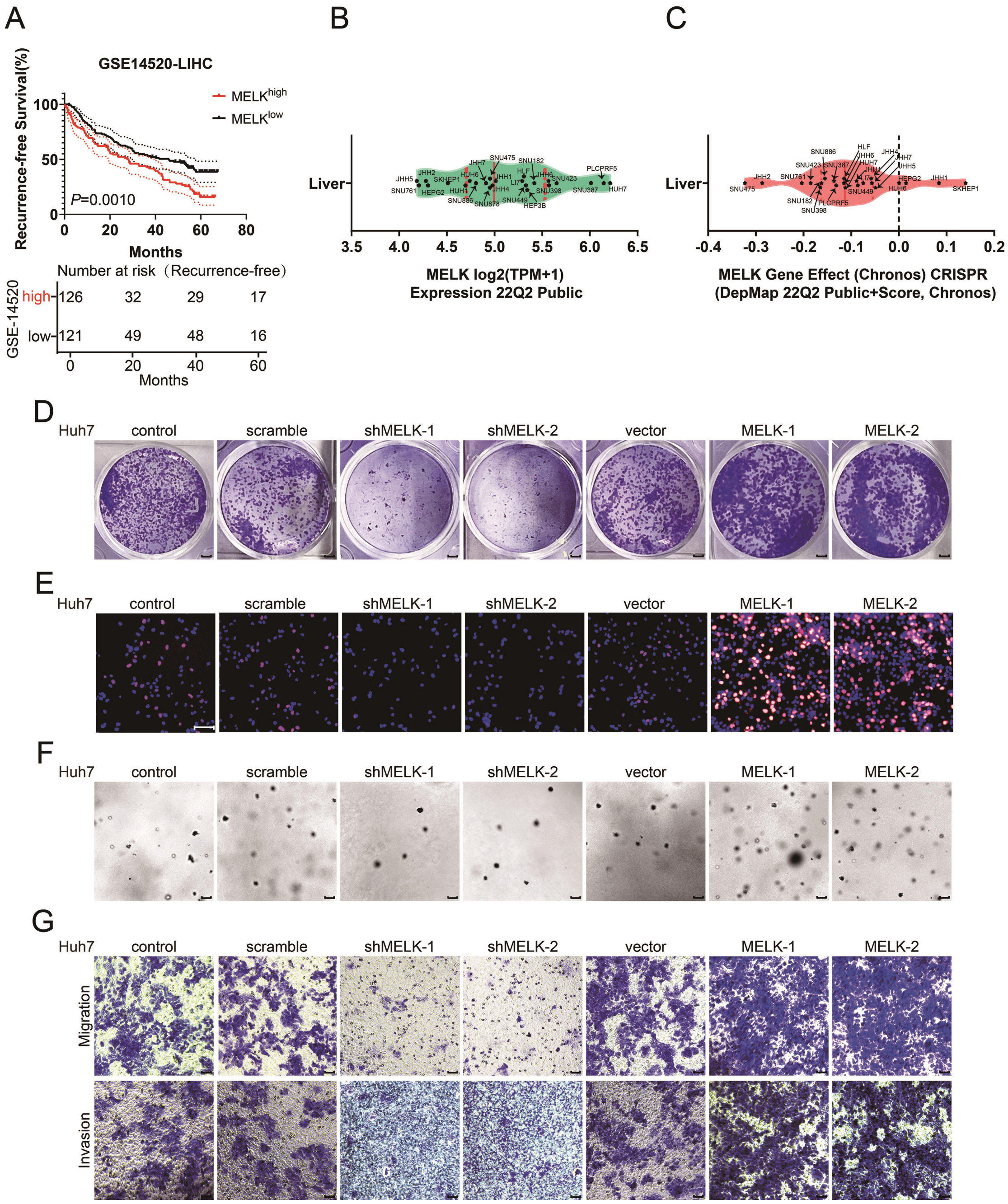

A The recurrence-free survival of MELK in GSE14520 cohorts. **B, C** The expression and gene effect of *MELK* in liver cancer cell lines in the Broad Institute DepMap web portal. **D** Representative images of colony formation in Figure 2G. **E** Representative images of EdU assay in Figure 2H. Scale bar: 100 μm. **F** Representative images of 3D cell sphere assay in Figure 2I. Scale bar: 200 μm. **G** Representative images of Transwell assay in Figure 2J, K. Scale bar: 50 μm.

Supplemental Figure S2. MELK was important in HCC progression

Supplemental-2

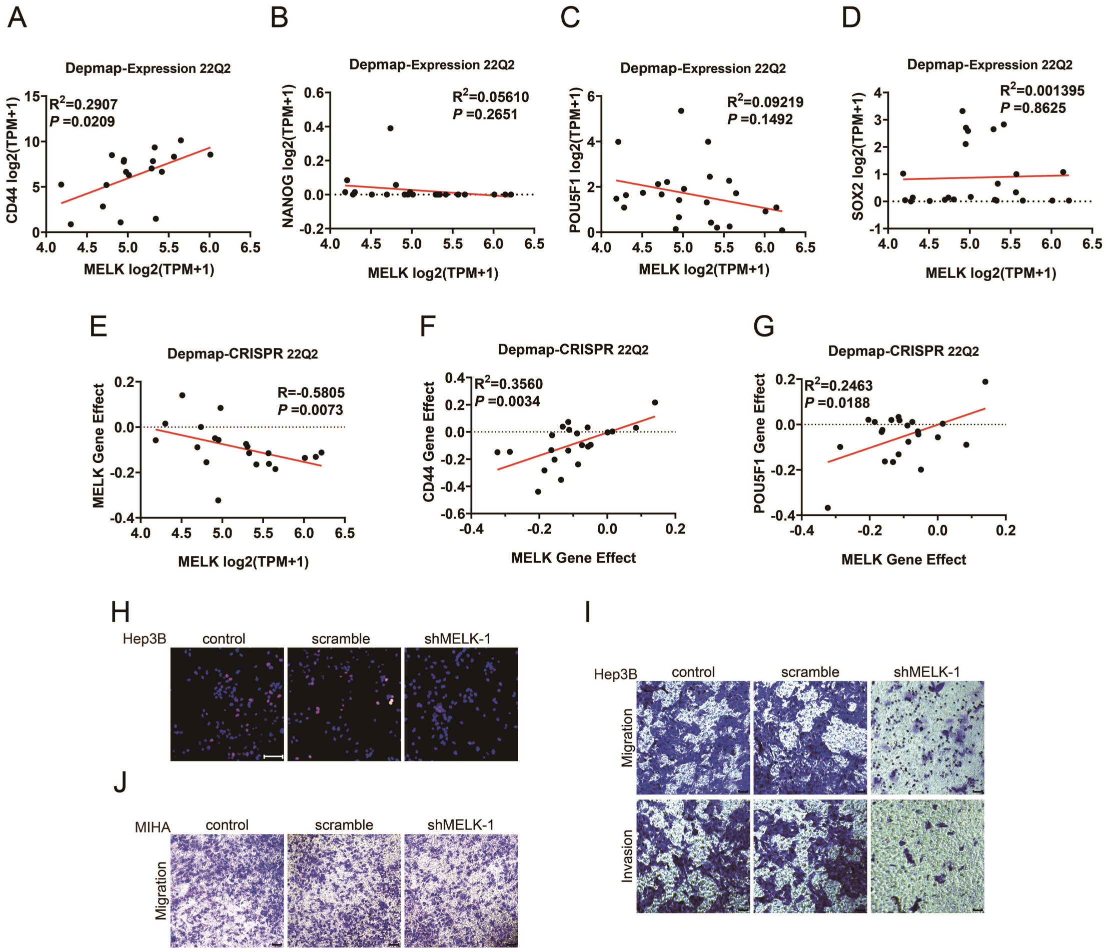

A-D Pearson correlation of *MELK* gene and stemness-related gene expression in HCC models. **E-G** Pearson correlation of *MELK* gene and stemness-related gene dependency in HCC models. **H** Representative images of EdU assay in Figure 2P. Scale bar: 100 μm. **I** Representative images of the Transwell assay in Figure 2Q. Scale bar: 50 μm. **J** Representative images of the Transwell assay in Figure 2T. Scale bar: 50 μm.

Supplemental Figure S3. Pearson correlation of *MELK* gene and candidate genes expression/dependency in HCC models

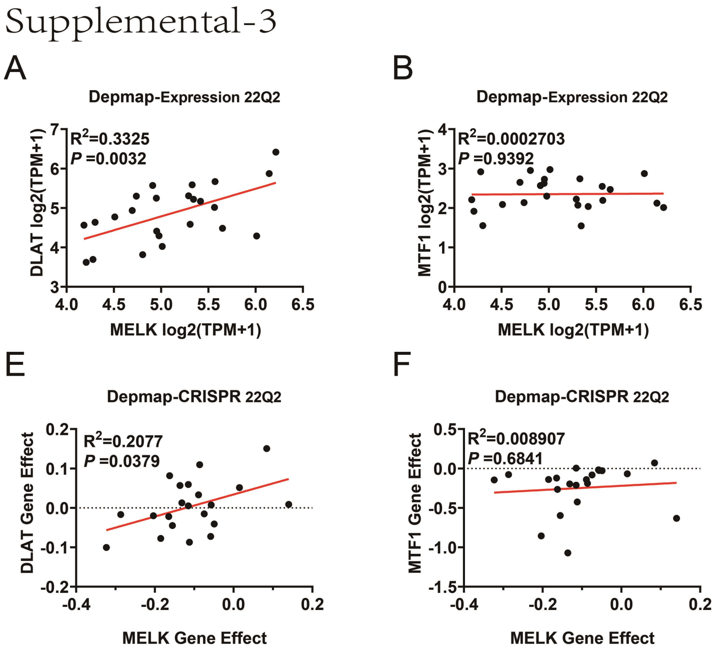

A-D Pearson correlation of *MELK* gene and candidate genes expression in HCC models. **E-H** Pearson correlation of *MELK* gene and candidate genes dependency in HCC models.

Supplemental Figure S4. MELK and DLAT demonstrate synergistic efficacy in HCC

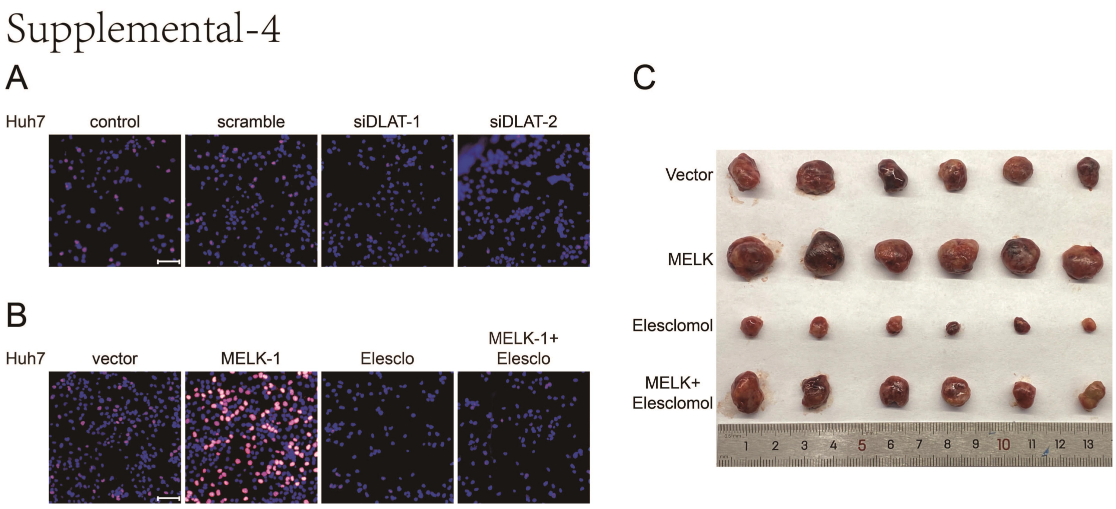

A Representative images of the EdU assay in Figure 6B. Scale bar: 100 μm. **B** Representative images of the EdU assay in Figure 6G. Scale bar: 100 μm. **C** Representative images of xenografts tumors in Figure 7A-C.

Supplementary Table S1 Relationships between MELK and clinicopathological features of hepatocellular carcinoma (HCC) patients in datasets of TCGA and GEO.

	TCGA-MELK	GSE 14520-MELK

		Low	High	P value	Low	High	P value
Age	<60	69	100	0.061	96	96	0.637
	≥60	104	101		23	27	
Gender	Male	125	128	0.096	103	108	0.848
	Female	48	73		16	15	
Fibrosis/Cirrhosis	Yes	66	74	0.253	108	115	0.480
	No	42	33		11	8	
AFP(ng/ml)	<=300	117	97	**0.000**	66	62	0.438
	>300	19	47		51	59	
Tumor Size (cm)	<=5				85	68	**0.011**
	>5				34	54	
Stage	I/II	128	132	**0.025**	95	79	**0.008**
	III/IV	32	58		17	34	
Child-Pugh	A	110	109	0.984			
	B/C	11	11				
BCLC	0/A				92	80	**0.045**
	B/C				20	33	
Histologic_grade	G1/G2	130	103	**0.000**			
	G3/G4	41	95				
BMI	<=26	91	114	0.265			
	>26	67	65				
Metastasis Risk	High				45	76	**0.000**
	Low				74	47	

Supplementary Table S2 Relationships between DLAT and clinicopathological features of hepatocellular carcinoma (HCC) patients.

		TCGA-DLAT			GSE 14520-DLAT		
		Low	High	P value	Low	High	P value
Age	<60	84	85	0.917	102	90	0.538
	≥60	100	105		29	21	
Gender	Male	117	136	0.122	108	103	**0.016**
	Female	67	54		23	8	
Fibrosis/Cirrhosis	Yes	72	68	0.650	119	104	0.411
	No	41	34		12	7	
AFP(ng/ml)	<=300	116	98	0.145	75	53	0.142
	>300	29	37		54	56	
Tumor Size (cm)	<=5				93	60	**0.008**
	>5				38	50	
Stage	I/II	141	119	**0.001**	101	73	**0.009**
	III/IV	31	59		19	32	
Child-Pugh	A	112	107	0.919			
	B/C	11	11				

BCLC	0/A				102	70	**0.001**
	B/C				18	35	
Histologic_grade	G1/G2	118	115	0.451			
	G3/G4	63	73				
BMI	<=26	105	100	0.451			
	>26	67	65				
Metastasis Risk	High				51	70	**0.000**
	Low				82	43	

Supplementary Table S3 Relationships between MELK/DLAT and clinicopathological features of hepatocellular carcinoma (HCC) patients in datasets of TMA.

		TMA-MELK			TMA-DLAT		
		Low	High	P value	Low	High	P value
Age	<60	32	25	0.070	27	30	0.563
	≥60	18	29		23	24	
Gender	Male	41	44	0.945	42	43	0.564
	Female	9	10		8	11	
Fibrosis/Cirrhosis	Yes	49	52	1.000	48	53	0.607
	No	1	2		2	1	
AFP(ng/ml)	<=300	39	30	**0.016**	38	31	**0.045**
	>300	11	24		12	23	
Tumor Size (cm)	<=5	35	26	**0.018**	36	25	**0.008**
	>5	15	28		14	29	
Stage	I/II	37	39	0.838	41	35	**0.048**
	III/IV	13	15		9	19	

Supplementary Table S4 The information of reagents and antibodies.

Reagent or resource	Source	Identifier
MELK	Proteintech Group	Cat # 11403-1-ap
DLAT	Abclonal	Cat # A14530
OCT4	Proteintech Group	Cat # 60242-1-lg
CD44	Proteintech Group	Cat # 60224-1-lg
EMT antibodies kit	Cell Signaling Technology	Cat # 9782 T
Caspase3	Abcam	Cat # ab32351
c-Caspase3	Abcam	Cat # ab32042
Caspase9	Cell Signaling Technology	Cat # 9508s
c-Caspase9	Cell Signaling Technology	Cat # 20750s
Bax	Abclonal	Cat # a19684
Bcl-2	Abclonal	Cat # a19693

HSP70	Abclonal	Cat # a12948
HSP90	Abclonal	Cat # a5027
TOM20	Beyotime	Cat # AF1717
PI3K	Cell Signaling Technology	Cat #84249T
p-PI3K	Cell Signaling Technology	Cat #4228S
AKT	Proteintech Group	Cat # 60203-2-lg
p-AKT	Proteintech Group	Cat # 66444-1-lg
mTOT	Proteintech Group	Cat # 66888-1-lg
p-mTOR	Proteintech Group	Cat # 67778-1-lg
β-actin	Cell Signaling Technology	Cat # 4970
Apoptosis Detection Kit	Beyotime	C1062M
EdU Cell Proliferation Kit	Beyotime	C0075L
ROS Assay Kit	Beyotime	S0033S
JC-1 Kit	Beyotime	C2006
Cell Mito Stress Test Kit	Agilent Technologies	103010-100
ATP Rate Assay Kit	Agilent Technologies	103591-100
Cell Copper (Cu) Colorimetric Assay Kit	Elabscience	E-BC-K775-M
740 Y-P	MedChemExpress	Cat # HY-P0175
PF-04691502	MedChemExpress	Cat # HY-15177
Elesclomol	MedChemExpress	STA-4783

Supplementary Table S5 Primers for qPCR.

Genes	Forward primer(5’-3’)	Reverse primer(5’-3’)
MELK	GCTTTCCTCACCTCCTGCAA	GCAATGCAGAGGTACCCGTT
DLAT	CTGGCTCACAAAGCAGAGGA	GCCAACCCAAGCAACTTCAG
GAPDH	GAGTCAACGGATTTGGTCGT	GACAAGCTTCCCGTTCTCAG

Suppementay Tabe S6 The related sequences of transfection.

Name	The shsiNA sequences
shMELK-1	CAGAAACAACAGGCAAACAAT
shMELK-2	GCCTGAAAGAAACTCCAATTA
Sh-srcambe –MELK	TTCTCCGAACGTGTCACGT
SiDLAT-1	CCAUACCUCAUUAUUACCUTT
SiDLAT-2	AGGUAAUAAUGAGGUAUGGTT
Scrambed DLAT	UUCUCCGAACGUGUCIACGUTT
